# Facial Localization of Malignant Chondroid Syringoma: A Rare Case Report

**DOI:** 10.1155/2013/907980

**Published:** 2013-02-13

**Authors:** Deniz Tural, Fatih Selçukbiricik, Feray Günver, Abdülkadir Karışmaz, Süheyla Serdengecti

**Affiliations:** ^1^Division of Medical Oncology, Department of Internal Medicine, Cerrahpasa Medical Faculty, Istanbul University, TR-34098 Istanbul, Turkey; ^2^Department of Pathology, Istanbul Education and Research Hospital, Istanbul, Turkey

## Abstract

First described by Hirsch and Helwig in 1961, chondroid syringomas (CSs) are rare, benign tumors of the skin arising from the eccrine sweat glands with tumor differentiation in the epithelial and mesenchymal tissues. They most commonly occur in the head and neck, although they may be also found in the axilla, trunk, limbs, and genitalia. The incidence of CS is <0.01% of all primary skin tumors. Malingnant chondroid syringomas (MCS), which are also called malignant mixed tumors of the skin, are extremely uncommon. MCSs commonly involve the limbs and rarely head and neck. In this article, we present a case of malignant chondroid syringoma localized in the face at the left nasolabial region in the light of literature review.

## 1. Introduction

Chondroid syringomas (CSs) are rare, benign tumors of the skin. In 1859, Billroth first described CS as a mixed tumor of the skin due to its histopathological resemblance to the benign mixed tumor originating from salivary gland [1, 2]. The term “chondroid syringoma” in lieu of mixed tumor of the skin was introduced by Hirsch and Helwig in 1961. They proposed the following microscopic diagnostic criteria: (a) nests of cuboidal or polygonal cells; (b) intercommunicating tubuloalveolar structures lined with two or more rows of cuboidal cells; (c) ductal structures composed of one or two rows of cuboidal cells; (d) occasional keratinous cysts; (e) a matrix of varying composition in hematoxylin and eosin stain [3]. In 1961, Headington divided CS into two groups, including apocrine type and eccrine type, based on their histopathological appearance [4]. 

Chondroid syringomas most commonly occur in the head and neck with a size ranging from 2 mm to ≥1 cm and usually present with solitary, solid, painless, nonulcerative, subcutaneous, or intracutaneous nodule [5, 6]. They often affect middle-age to elderly patients with a male-to-female ratio of 2 : 1 [6–8].

The malignant variant of CS is rare. In most cases of MCS, anaplastic changes are present from the beginning. Malignant chondroid syringoma is a mixed cutaneous tumor, with epithelial and mesenchymal components, which compromises principally the trunk and extremities. Rarely, a chondroid syringoma of many years duration suddenly undergoes malignant changes with widespread metastasis [9].

There have been no reports reporting effectiveness of chemotherapy and radiotherapy, and an early wide excision with a broad margin may be the most reliable treatment to date.

In this paper, we present a case of MCS localized in the face at the left nasolabial region in the light of literature review.

## 2. Case Report

**Figure 1:**
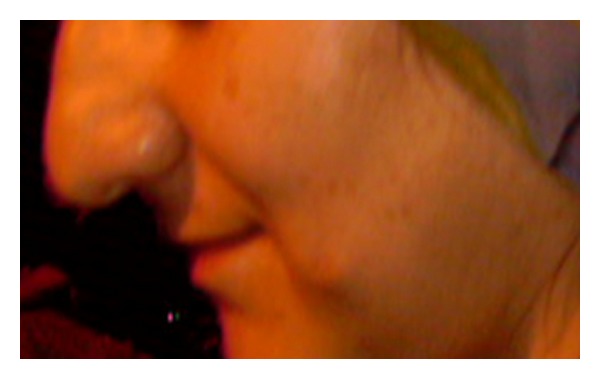
A 34-year-old female was admitted with a slowly growing nodular lesion at the left nasolabial region for three years.

**Figure 2:**
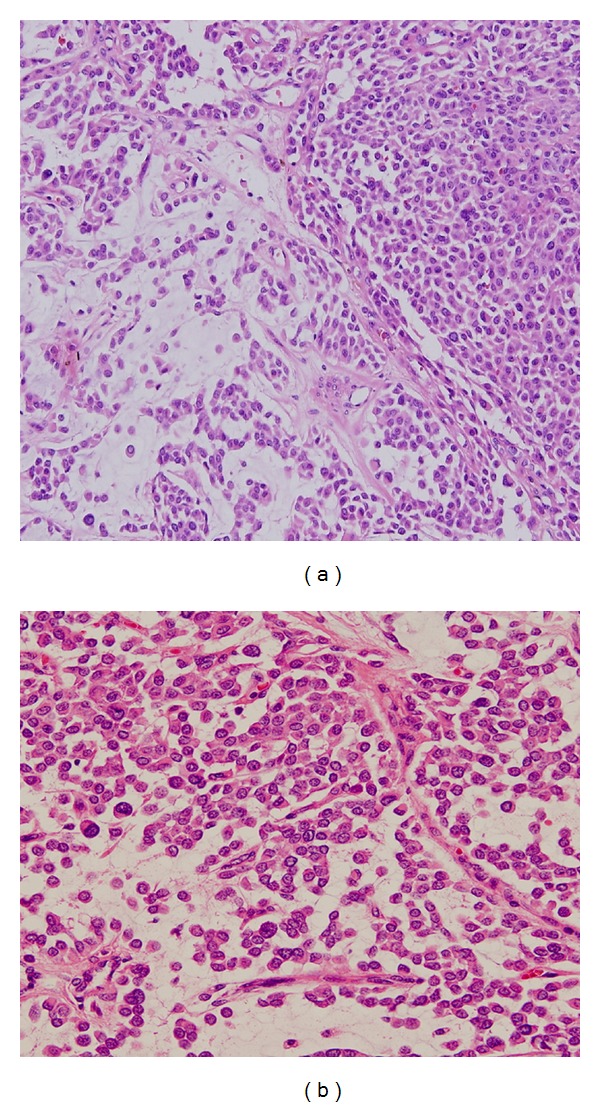
Histopathological examination of nasolabially excised mass. Tumor had a benign nature mostly, with an expansive growing pattern and a few pleomorphic, atypical cells, and rare mitotic activity. In addition, generalized lymph vascular invasion was present ((a) HE, ×100; (b) HE, ×200).

A 34-year-old female was admitted with a slowly growing nodular lesion at the left nasolabial region for three years (Figure 1). Excisional biopsy revealed neurofibroma. The patient had been excised earlier; however, recurrent lesion was present in the first year of the procedure. Then, the patient underwent wide excision at the left nasolabial region. Based on the pathological examination, the patient was diagnosed with MCS. Physical examination also revealed a single palpable lymphadenopathy at the left side of the neck. The patient underwent functional left neck dissection, and pathological examination of the excised material showed no metastatic disease. Histopathological examination of nasolabially excised mass revealed that the tumor had an expansive growing pattern and a few pleomorphic, atypical cells, and rare mitotic activity. In addition, generalized lymphovascular invasion was present (Figures 2(a) and 2(b)). No other tumor at surgical margin was found with a nearest margin of 1 cm. Complete blood count and regular biochemistry parameters were normal. Thoracic, abdominal, and cranial computed tomography (CT) scans that were performed to detect any systemic involvement indicated no metastatic disease. The patient, who has been disease-free for two years following surgery, is still under followup every three months for any recurrence. 

## 3. Discussion

Chondroid syringomas are tumors arising from sebaceous glands, sweat glands, and ectopic salivary glands [10]. They usually present with slow-growing, painless, solid, subcutaneous or intradermal nodules with a normal margin. They account for <0.01% of all primary skin tumors. They most commonly occur in the head and neck of adult males, followed by nose, cheeks, upper lip, scalp, forehead, and chin. Tumor size may range from 2 mm to ≥1 cm [5–8]. The lesion is usually solitary with a benign nature. However, very rare malignant cases were also reported. 

Analysis of the reported cases showed that the mean age of the patients at the time of diagnosis was 48.3 years (range 13–84 years). The neoplasm tends to affect limbs mostly (61% in limbs, 17% in trunk, and 22% in head and neck), while its benign counterpart occurs mainly in the head and neck (80% in head and neck, 10% in limbs, and 10% in trunk) [11, 12]. The female-to-male ratios are 3 : 2 in MCS and 2 : 7 in its benign counterpart [11]. 

In our case, the patient had a history of a slowly growing lesion at the left nasolabial region and recurrent lesion within the first year of following local excision with a size of 1.5 cm. Malignant chondroid syringoma may occur de novo or rarely develop in a chondroid syringoma. In contrast to the benign counterpart, which is common in the head and neck region, the malignant variety occurs predominantly on the trunk and extremities [13–15]. In our patient, a middle-aged woman, the tumor was on left labial sulcus of the face and recurred after previous excision. The female preponderance of this tumor is reported in many studies [14–17]. 

In our case also, prior studies that had mitosis, nuclear atypia, pleomorphism, lymphatic invasion, and local recurrence have been recognized as helpful signs for the diagnosis of malignancy [9, 14–16, 18]. Chondroid syringoma may be confused clinically with epidermal cyst, pilar cyst, calcifying epithelioma, or a solitary trichoepithelioma [19]. In our case, the tumor was initially diagnosed as neurofibroma. The definitive diagnosis was based on the wide excision of the tumor which recurred following the initial excision with a rapid growing pattern, which is probably the reason for the tumor not being sent for histopathology after excision earlier. Recurrence of the lesion alerts the clinician to the possibility of malignancy [16, 19]. 

This highlights the need for microscopic diagnosis of all tumors that are excised irrespective of the clinical diagnosis. Treatment consists of complete excision of the tumor. Though local radiotherapy is often unsuccessful, skeletal metastasis has been shown to respond to radiotherapy [16]. Combination chemotherapy in patients with metastasis is not reported to be beneficial [16]. Adequate surgical excision with wide disease-free margins is the only hope for disease control [14, 16, 20]. Our case has been defined as disease-free for two years following wide excision. 

In terms of prognosis of the reported cases, 8 of the 30 (27%) patients died from their disease. Death occurred as early as 9 weeks following surgery; one patient survived 12 years after diagnosis [10, 21]. 

Malignant chondroid syringoma tends to follow an unpredictable clinical course. Of the reported cases, 50% had local recurrences [20–22]. Nodal metastases and distant metastases were observed in 39% and 36% of the cases, respectively. The most common site for distance metastasis was lung, followed by bone and brain [20, 23].

In conclusion, negative surgical margin should be attained using wide excision technique. These patients should be also monitored closely due to high potential of recurrence.
